# Serial interferon-gamma release assays during treatment of active tuberculosis in young adults

**DOI:** 10.1186/1471-2334-10-300

**Published:** 2010-10-16

**Authors:** Sei Won Lee, Choon-Taek Lee, Jae-Joon Yim

**Affiliations:** 1 Department of Internal Medicine, Armed Forces Capital Hospital, Seongnam, Republic of Korea; 2 Department of Internal Medicine, Seoul National University Bundang Hospital, Seongnam, Republic of Korea; 3 Division of Pulmonary and Critical Care Medicine, Department of Internal Medicine and Lung Institute, Seoul National University College of Medicine, Seoul, Republic of Korea

## Abstract

**Background:**

The role of interferon-γ release assay (IGRA) in monitoring responses to anti-tuberculosis (TB) treatment is not clear. We evaluated the results of the QuantiFERON-TB Gold In-tube (QFT-GIT) assay over time during the anti-TB treatment of adults with no underlying disease.

**Methods:**

We enrolled soldiers who were newly diagnosed with active TB and admitted to the central referral military hospital in South Korea between May 1, 2008 and September 30, 2009. For each participant, we preformed QFT-GIT assay before treatment (baseline) and at 1, 3, and 6 months after initiating anti-TB medication.

**Results:**

Of 67 eligible patients, 59 (88.1%) completed the study protocol. All participants were males who were human immunodeficiency virus (HIV)-negative and had no chronic diseases. Their median age was 21 years (range, 20-48). Initially, 57 (96.6%) patients had positive QFT-GIT results, and 53 (89.8%), 42 (71.2%), and 39 (66.1%) had positive QFT-GIT results at 1, 3, and 6 months, respectively. The IFN-γ level at baseline was 5.31 ± 5.34 IU/ml, and the levels at 1, 3, and 6 months were 3.95 ± 4.30, 1.82 ± 2.14, and 1.50 ± 2.12 IU/ml, respectively. All patients had clinical and radiologic improvements after treatment and were cured. A lower IFN-γ level, C-reactive protein ≥ 3 mg/dl, and the presence of fever (≥ 38.3°C) at diagnosis were associated with negative reversion of the QFT-GIT assay.

**Conclusion:**

Although the IFN-γ level measured by QFT-GIT assay decreased after successful anti-TB treatment in most participants, less than half of them exhibited QFT-GIT reversion. Thus, the reversion to negativity of the QFT-GIT assay may not be a good surrogate for treatment response in otherwise healthy young patients with TB.

## Background

Tuberculosis (TB) remains one of the most significant infectious diseases worldwide. Despite intensive global efforts, the number of new TB cases continues to increase, with 9.27 million new cases and 1.78 million deaths in 2006 [1]. Rapid diagnosis and appropriate treatment of TB are important not only for the health of individuals but also for effective disease control in a population. To evaluate treatment efficacy, sputum microscopy is still used, despite its reportedly low sensitivity [2].

The interferon-gamma release assay (IGRA) has recently been adopted for detecting TB infection [3]. Its role in the diagnosis of latent TB infection (LTBI) is promising [4], and it has some advantages over the tuberculin skin test (TST): it is not affected by previous Bacille Calmette-Guérin (BCG) vaccination, it minimizes technical errors, and it has higher specificity[5]. However, its application in assessing treatment efficacy is not clear [6]. Several reports have suggested that the IGRA is useful for evaluating the efficacy of treatment for active TB, although most of the studies enrolled a small number of patients or those with various immune statuses [7-10].

To examine the potential of the IGRA as a surrogate for treatment response among young immunocompetent patients with TB, we evaluated the sequential changes in the interferon-gamma (IFN-γ) level measured by the QuantiFERON-TB Gold In-tube (QFT-GIT; Cellestis, Victoria, Australia) assay in immunocompetent adults with TB, but with no underlying disease.

## Methods

### Participants and data collection

All soldiers who were diagnosed with active TB between May 2008 and September 2009 at the Armed Forces Capital Hospital, a central referral military hospital in South Korea, were eligible for this study. Those who could not be followed for at least 6 months or who received anti-TB medication before admission to our hospital were excluded. After giving informed consent, each participant completed a questionnaire regarding their demographics, history of TB, smoking status, and other factors. We performed QFT-GIT assay for each participant before treatment (baseline) and after 1, 3, and 6 months of anti-TB treatment. This study was reviewed and approved by the Institutional Review Board of the Armed Forces Medical Command.

### Diagnosis and treatment of TB

Active pulmonary TB was diagnosed when (1) the patient's sputum was positive for acid-fast staining, or *Mycobacterium tuberculosis *was cultured; or (2) the patient's data met the definition of a clinical case of TB according to the World Health Organization criteria [11]. To diagnose clinical TB, respiratory symptoms and radiographic lesions on high-resolution computed tomography (HRCT) that were compatible with active TB were mandatory. Active pulmonary TB was defined as the presence of cavities, branching linear opacities, or multiple non-calcified nodules on HRCT [12-14]. For participants with lesions that suggested active TB, but who did not have positive acid-fast smears or *M. tuberculosis *cultures, broad-spectrum antibiotics (a combination of a third-generation cephalosporin and a macrolide) were prescribed for 1 week; if the lesions on chest X-ray or HRCT showed a definite improvement, a diagnosis of active TB was excluded.

TB pleuritis was diagnosed when (1) *M. tuberculosis *was detected in a culture of pleural fluid or tissue; or (2) positive real-time polymerase chain reaction for *M. tuberculosis *in pleural fluid or tissue; or (3) an exudative pleural effusion showed lymphocyte predominance, negative cytology, low carcinoembryonic antigen (< 5 ng/ml), and high adenosine deaminase (≥ 50 IU/L).

TB lymphadenitis was diagnosed when one of the following criteria was met: (1) cultivation of *M. tuberculosis *from lymph node tissue; (2) presence of a caseating granuloma with or without visible acid-fast bacilli; or (3) positive real-time polymerase chain reaction for *M. tuberculosis *in lymph node tissue.

All patients were given standardized anti-TB medication consisting of daily isoniazid (INH), rifampicin (RMP), ethambutol (EMB), and pyrazinamide (PZA) for 2 months, followed by daily INH, RMP, and EMB for 4 months [15]. In cases with INH resistance, daily RMP, EMB, PZA, and a fluoroquinolone (levofloxacin or moxifloxacin) were given for at least 6 months.

### Tuberculin skin test

After the initial blood sampling for the QFT-GIT assay, a TST was performed by trained personnel following standard procedures. For this test, 0.1 ml (2 TU) of purified protein derivate (RT23, Statens Serum Institute, Copenhagen, Denmark) was injected intradermally into the inner side of the left forearm. The transverse induration at the TST site was measured in millimeters after 48-72 h, by one of three experienced nurses. We defined a positive test as one in which there was induration ≥ 10 mm [16].

### Interferon-gamma release assay

The QFT-GIT was performed according to the manufacturer's instructions. Blood was collected in three special tubes: one coated with the *Mycobacterium-tuberculosis*-specific peptides ESAT-6, CFP-10, and TB 7.7 (Rv2654, only peptide 4); one coated with mitogen, as a positive control; and one without antigen coating, as a negative control. Within 8 h of blood sampling, the tubes were incubated for 24 h at 37°C, centrifuged, and stored in the cold until testing, as specified by the manufacturer. The plasma IFN-γ concentration was measured using a commercial QFT-GIT ELISA and was determined as negative, intermediate, or positive (cut-off at 0.35 IU/ml) by the manufacturer's software. QFT-GIT reversion to negativity was defined as a change in the IFN-γ level from a positive (≥ 0.35 IU/ml) to a negative result (< 0.35 IU/ml). Because the QFT-GIT ELISA cannot measure IFN-γ values > 10 IU/ml [7] accurately, values > 10 IU/ml were treated as 10 IU/ml.

### Statistical analysis

Agreement between the TST and QFT-GIT results was measured using the kappa statistic. McNemar's test was used to compare paired proportions of QFT-GIT-positive results. Repeated measures analysis of variance was used to compare the IFN-γ levels at different time points, and the results were validated using Mauchly's test of sphericity (equal variance of the differences between levels of the repeated measures factor). The means of different groups were compared using Student's *t*-test. The relationship between clinical characteristics and QFT-GIT reversion to negativity was evaluated using the χ^2 ^test. Statistical significance was assessed at a two-tailed *P *value of 0.05, and all data are presented as the mean ± standard deviation except figure 3. All statistical analyses were conducted using PASW 17.0 (SPSS, Chicago, IL, USA).

## Results

### Demographic and clinical characteristics of the participants

Sixty-seven soldiers with active TB were eligible for this study; eight were lost to follow-up. Thus, 59 (87.9%) participants completed the study protocol and were included in the final analysis. All participants were male and had a median age of 21 years (range, 20-48). Every participant was negative for human immunodeficiency virus (HIV) and had no other underlying disease (e.g. uncontrolled asthma, diabetes mellitus, and chronic renal failure). Forty-six (78.0%) patients had pulmonary TB: 13 (28.9%) had positive acid-fast staining of their sputum, and 33 (71.7%) had *M. tuberculosis *cultured from their sputum. In 29 of these 33 participants, the isolates were sensitive to all anti-TB medications; the isolates from four patients showed INH mono-resistance. Patients with other types of TB included 11 with pleural TB (including one patient with culture-positive pleural fluid), one with TB lymphadenitis, and one with a TB abscess in the inguinal area (Table 1). All 11 patients with pleural TB and the one patient with a TB abscess had pulmonary parenchymal involvement[17]. All patients in the study had radiologic and clinical improvements, with negative conversion in a sputum exam at 2 months after anti-TB treatment. None of the 59 patients had a relapse of active TB during the follow-up period of 102 ± 105 days (range, 0-433).

**Table 1:** Demographic and clinical characteristics of the participants included in the final analysis

Characteristics	Values (n = 59)
Median age: years (range)	21 (20-48)
Male: n (%)	59 (100)
BCG scar present: n (%)	44 (74.6)
Current smoker	26 (44.1)
Diagnosis	
Pulmonary TB (%)	46 (78.0)
Smear positive	13 (28.9)*
Culture positive	33 (71.7)*
TB pleuritis	11 (18.6)
TB lymphadenitis	1 (1.7)
TB abscess	1 (1.7)

* The percentages were calculated using participants with pulmonary TB as the denominator.

### Changes in QFT-GIT during anti-TB treatment

**Figure 1:**
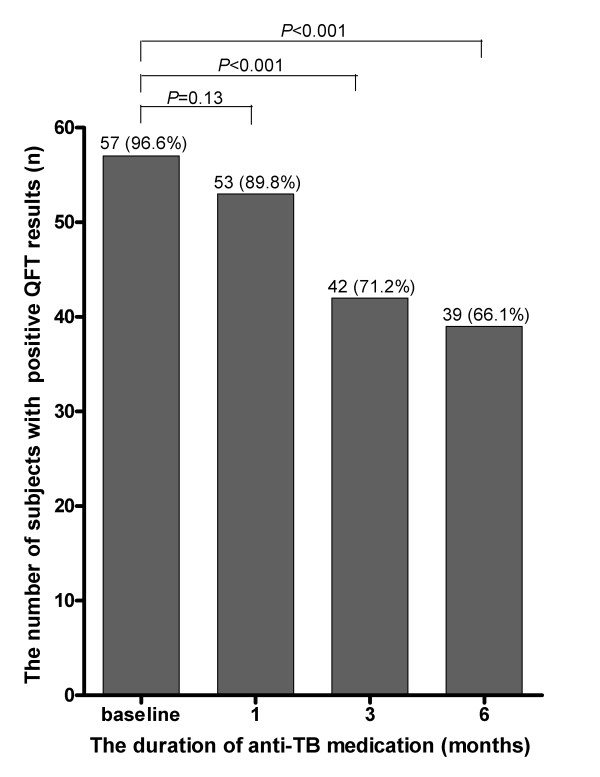
**The proportion of active tuberculosis patients with positive QFT-GIT results**.

Fifty-seven (96.6%) patients had positive QFT-GIT results and 56 (94.9%) had positive TST results at the diagnosis of TB. The agreement between the two tests was substantial (kappa = 0.79, *P *< 0.001) [18], with an agreement rate of 98.3%. After treatment, 53 (89.8%) had a positive QFT-GIT result at 1 month; 42 (71.2%), at 3 months; and 39 (66.1%), at 6 months. The proportion of patients with a positive QFT-GIT result decreased significantly at 3 months (*P *< 0.001; Figure 1).

**Figure 2:**
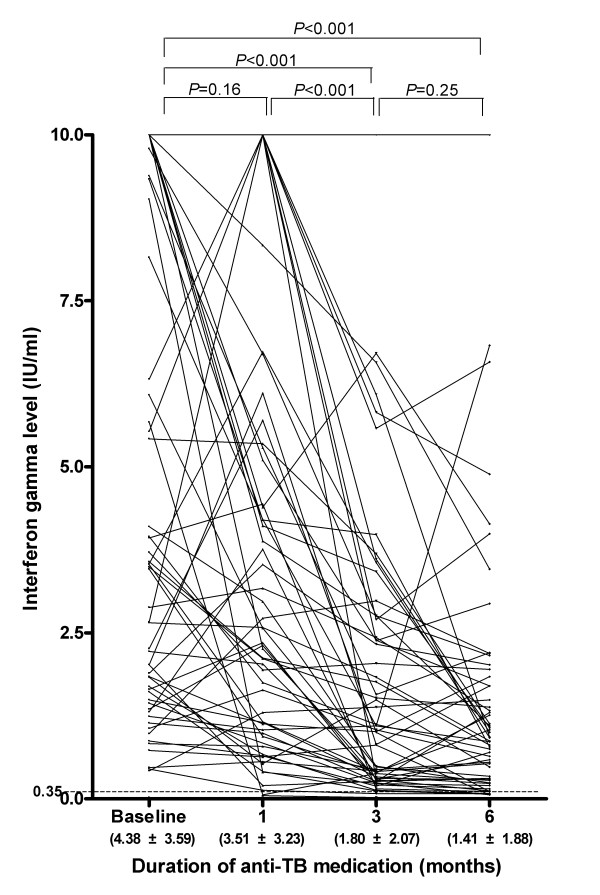
**The change in the IFN-γ level during successful anti-TB treatment**.

**Figure 3:**
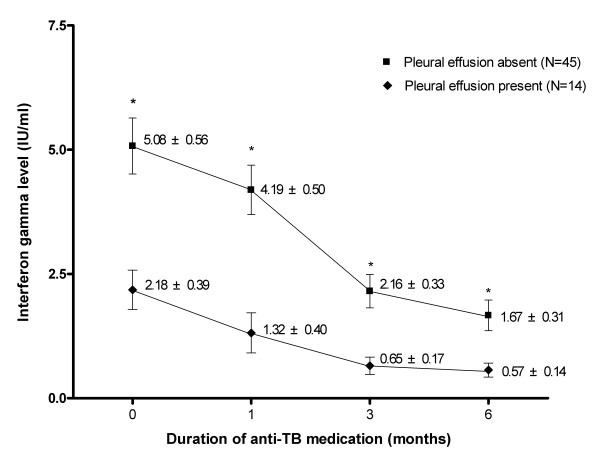
**Comparison of changes in the IFN-γ level between patients with pulmonary tuberculosis according to the presence of pleural effusion.**  Data were presented as mean ± standard error.  * P<0.01 (between two groups)

The IFN-γ level at baseline was 4.38 ± 3.59 IU/ml, and the levels at 1, 3, and 6 months were 3.51 ± 3.23 IU/ml, 1.80 ± 2.07 IU/ml, and 1.41 ± 1.88 IU/ml, respectively. The IFN-γ level decreased with time, with a significant decrease at 3 months (*P *< 0.001), but not at 1 month (*P *= 0.16). The IFN-γ level did not differ significantly between 3 and 6 months (*P *= 0.25, Figure 2). The IFN-γ level decreased during treatment in 55 (93.2%) participants. The mean difference in the IFN-γ level between baseline and 6 months was -3.75 ± 4.60 IU/ml, and the ratio of the IFN-γ level at 6 months to that at baseline was 0.41 ± 0.58. The IFN-γ level at 6 months was less than half of the baseline level in 43 (72.9%) participants (Table 2). Throughout the treatment, the IFN-γ levels had been lower among TB patients with pleural effusion than among patients without pleural effusion. (Figure 3)

**Table 2:** Difference and proportion of IFN-γ levels at 6 months compared with those at baseline

Interferon-gamma level	Number of participants (%/cumulative %)
Difference between baseline and 6 months (IU/ml)	
≤ -8	7 (11.9/11.9)
> -8 and ≤ -5	9 (15.3/27.1)
> -5 and ≤ -2	11 (18.6/45.8)
> -2 and ≤ -1	14 (23.7/69.5)
> -1 and ≤ 0	15 (25.4/94.9)
> 0 and < 1.3	3 (5.1/100.0)

Proportion of participants with defined change (%)	
≤ 10	15 (25.4/25.4)
> 10 and ≤ 25	14 (23.7/49.2)
> 25 and ≤ 50	13 (22.0/71.2)
> 50 and ≤ 100	14 (23.7/94.9)
> 100 and ≤ 360	3 (5.1/100.0)

### Participants with unusual change of IFN-γ response

Three participants showed increased IFN-γ levels after treatment, with levels increasing from 1.89 to 2.02, 0.42 to 1.49, and 0.98 to 2.19 IU/ml, respectively. In one participant, the IFN-γ level remained at 1.87 IU/ml. Two patients with an initial negative QFT-GIT result remained persistently QFT-GIT negative for 6 months (Figure 2).

One subjects with TB lymphadenitis had an IFN-γ level of > 10.0 IU/ml persistently during 6 months of treatment and he was the only participant whose IFN-γ level exceeded 10 IU/ml after completing therapy. His neck lymph node had a longest diameter of 4 cm at baseline, but was undetectable at treatment completion, and there was no evidence of relapse for 6 months after treatment completion.

### Characteristics associated with negative conversion of the QFT-GIT

C-reactive protein (CRP) level higher than 3 mg/ml (*P *= 0.046) and body temperature above 38.3°C (*P *= 0.056) at diagnosis were associated with reversion to negativity of the QFT-GIT, as was a lower baseline IFN-γ level (*P *= 0.03, Table 3).

**Table 3:** Variables associated with QFT-GIT reversion

	Number (%)	Number with reversion (%)	Odds ratio (95%CI)	*P *value
Cavity				
Present	18 (31.6)	6 (33.3)	1.00 (reference)	0.85
Absent	39 (68.4)	12 (30.8)	1.13 (0.34-3.71)	
Lesion extent				
> one lobe	28 (49.1)	8 (27.6)	1.00 (reference)	0.45
one lobe	29 (50.9)	10 (35.7)	1.55 (0.50-4.77)	
Pleural effusion				
Absent	43 (75.4)	11 (25.6)	1.00 (reference)	0.10
Present	14 (24.6)	7 (50.0)	2.91 (0.83-10.17)	
CRP				
< 3 mg/dl	41 (73.3)	10 (24.4)	1.00 (reference)	0.046
≥ 3 mg/dl	15 (26.8)	8 (53.3)	3.54 (1.03-12.24)	
Body temperature*				
< 38.3°C	44 (76.8)	11 (25.6)	1.00 (reference)	0.056
≥ 38.3 °C	13 (23.2)	7 (53.8)	3.50 (0.97-12.67)	
Baseline QFT-GIT				
decrease by 1 IU/ml	57 (100)	18 (31.6)	1.24 (1.03-1.49)	0.03

All analysis was performed with exclusion of two participants with initial negative QFT.

*Peak body temperature within 24 hours of initial hospital visit.

QFT-GIT, QuantiFERON-TB gold in-tube assay; CRP, C-reactive protein.

## Discussion

This study reports the changes over time in the IFN-γ levels of young active TB patients with no underlying disease, during treatment with anti-TB medication. All 59 patients were cured; however, only 18 (32.1%) of the 56 participants with an initial positive QFT-GIT result showed reversion to negativity after 6 months of anti-TB medication. With 6 months of treatment, the IFN-γ level decreased to less than half of the baseline level in 43 (72.9%) participants.

A preliminary report suggested that the IGRA was useful for monitoring the efficacy of anti-TB therapy in patients with active TB [19]. However, subsequent studies reported discrepant results [3]. Longitudinal studies using a QuantiFERON-TB Gold (QFT-G) assay gave variable results. Two studies conducted in Japan showed a progressive decrease of the IFN-γ response after anti-TB treatment, with smaller percentages of subjects with positive QFT-G results over time [20,21]. By contrast, a study conducted in India showed a persistent IFN-γ response during treatment, with positive QFT-G rates of 73% at baseline and 79% at 6 months, although the average IFN-γ level declined slightly [7]. This discrepancy may be related to the initial IFN-γ levels and the burden of TB in the countries. The baseline IFN-γ level in the study conducted in India was 4.09 ± 4.14 IU/ml, which was greater than those in the two studies from Japan (0.51 ± 0.15 and 0.75 ± 0.15 IU/ml), and a higher IFN-γ level was reported to be associated with a lower likelihood of converting to a negative culture [10]. Furthermore, in high-burden countries, ongoing exposure and exogenous re-infection can keep T-cell responses strong, even after the antigen load has declined with therapy.

The lower reversion rate after completing anti-TB treatment (31.6%) in our study could be explained by the fact that the initial IFN-γ level (4.38 ± 3.59 IU/ml) was higher than those in the previous studies in Japan. The higher initial IFN-γ level in our study may be associated with the demographic characteristics of the participants, who were young, immunocompetent patients with no underlying disease. To achieve reversion to negativity in patients with a high initial IFN-γ level, the absolute decrease must be profound.

In addition to low baseline IFN-γ level, higher CRP level and the presence of fever were also associated with the reversion to negativity of QFT-GIT in this study. The marginal statistical significance (*P *= 0.056) of the association between fever and reversion to negativity may be due to the small number of participants. IL-12-dependent IFN-γ release is crucial for controlling TB infection [22,23]. Therefore, relatively weak immune responses with lower IFN-γ release may be related to a severe initial infectious state, presenting as fever and a high CRP level. Reversion to negativity of the QFT-GIT in these patients may be achieved easily because of the lower initial IFN-γ level.

Interestingly, TB patients with pleural effusion had consistently lower IFN- γ levels than those without pleural effusion throughout the treatment period. Considering the fact that the development of tuberculous pleural effusion is suggested to be based on delayed type hypersensitivity reaction [24,25], lower IFN- γ levels among TB patients with effusion was somewhat unexpected. This observation should be confirmed in future studies involving larger number of patients.

In this study, 96.6% of the participants with active TB had positive results on the first QFT-GIT; this rate is higher than previously reported rates. With the exception of two studies from South Korea [26] and Japan [20], most studies reported low sensitivity (64-76%) with this assay[27-31]. The higher sensitivity of the assay in our study could be explained by the characteristics of the participants, who were mostly young, otherwise healthy, immunocompetent military personnel. The sensitivity of the IGRA is known to decrease significantly with older age [32], HIV infection [33], and chronic renal failure [34].

## Conclusion

Although the IFN-γ levels in the QFT-GIT decreased after successful anti-TB treatment in most participants, less than half of them had reversion to negativity of QFT-GIT among young immunocompetent patients with active TB. The reversion of the QFT-GIT with anti-TB medication was more common among patients with a lower initial IFN-γ level, higher CRP, and fever at the diagnosis of active TB.

## Competing interests

The authors declare that they have no competing interests.

## Authors' contributions

SWL and JJY planned this study, collected and analyzed the data, and wrote the manuscript. CTL supervised this study. All authors read and approved the final manuscript.

## Pre-publication history

The pre-publication history for this paper can be accessed here:

http://www.biomedcentral.com/1471-2334/10/300/prepub
